# Accelerated intimal hyperplasia in aortocoronary internal mammary vein grafts in minipigs

**DOI:** 10.1186/1749-8090-3-20

**Published:** 2008-04-29

**Authors:** Aron Frederik Popov, Hilmar Dorge, Jose Hinz, Jan Dieter Schmitto, Tomislav Stojanovic, Ralf Seipelt, Vassilios Didilis, Friedrich Albert Schoendube

**Affiliations:** 1 Department of Thoracic Cardiovascular Surgery, University of Göttingen, Germany; 2 Department of Anaesthesiology, Emergency and Intensive Care Medicine, University of Göttingen, Germany; 3 Department of Cardiothoracic surgery, Democritus University of Thrace, Greece

## Abstract

**Background:**

More than 50% of aortocoronary saphenous vein grafts are occluded 10 years after surgery. Intimal hyperplasia is the initial critical step in the progression toward occlusion. Internal mammary veins, which are physiologically prone to less hydrostatic pressure, may undergo an accelerated progression to intimal hyperplasia and thus be suitable for investigation of the mechanisms of aortocoronary vein graft disease.

**Methods:**

Six minipigs underwent aortocoronary bypass grafting using standard cardiopulmonary bypass and cardioplegic arrest. Mammary vein were grafted in a reversed manner from ascending aorta to left anterior descending coronary artery (LAD). The proximal LAD was ligated, rendering the anterior left ventricle vein graft-dependent. Minipigs were killed after 4 weeks, and vein grafts were harvested. Histological and immunohistological investigation were performed with respect to morphometric analysis, endothelial damage/dysfunction (v-Willebrand-factor (vWF)), smooth muscle cells (α-smooth actin) and proliferation rate (proliferation marker Ki 67).

**Results:**

Mean intimal area of vein grafts was increased compared to ungrafted mammary veins. Intimal hyperplasia in vein grafts was characterized by massive accumulation of smooth muscle cells with a high proliferation rate and endothelial perturbation. Significant (*p *= 0.001) intimal hyperplasia of the grafted mammary vein compared to the ungrafted mammary vein was found. These changes were absent in ungrafted mammary veins.

**Conclusion:**

The present study demonstrates a pig model of aortocoronary vein graft intimal hyperplasia which is characterized by an accelerated progression within internal mammary veins. The model is suitable to investigate the pathophysiology of aortocoronary vein graft intimal hyperplasia as well as therapeutic approaches.

## Introduction

The saphenous vein is still a conduit of choice for coronary artery bypass grafting. Following arterializations, vein grafts undergo immediate injury like ischemia and wall stress. The histological changes associated with vein graft failure are defined as intimal hyperplasia. They include acute thrombosis or early medial and intimal thickening that may be focally progressive. Further reason is late artheroma formation, which is the most important cause of failure beyond five years after implantation [1]. This vein graft failure is result of progressive thickening of the intima and media acting over the first months and slow process of atherosclerosis over years [2]. According to this, major limitation for its use is the high graft occlusion rate, which increase from 8% early on 14^th ^day, to 13% at 1 year up to 20% at five years after operation [3].

Vein graft failure and intimal hyperplasia have already been shown in large animals, like dogs and pigs [4-8]. The advantage of large animal is the more human like wall of the vein graft compared with veins of small animal. Because of their size breeds of 50 kilogram are sufficient for standard arthrosclerosis long term research. However, these breeds can gain up to 1 kilogram per day and reach weights of more than 200 kilogram and are difficult to handle under laboratory conditions. The anatomical structures of these pigs are not suited for standard interventional cardiac techniques, and equipment used for human procedures can not be easily applied. After six month the Goettingen minipig reached a body weight of 50 kilogram and gain up no more. The anatomical conditions like size, structure and functions of the heart of the Goettingen minipig approach those of humans allowing a transfer of the animal results obtained. The size of veins and coronary artery in these pigs are ideal for coronary artery surgery [9].

Because of the great differences in hydrostatic pressure acing along the blood vessels in erect posture, saphenous vein is exposed to greater transmural pressures than internal mammaria vein. Internal mammaria vein may therefore suitable for an accelerated model of intimal hyperplasia and atherosclerosis.

In the present study we used a minipig model and internal mammary vein for aortocoronary bypass surgery. Internal mammaria vein were grafted in reversed manner from the ascending aorta to the left anterior descending coronary artery followed from ligating the proximal left anterior descending coronary. Our objective was to establish a model for accelerated intimal hyperplasia within four weeks in aortocoronary internal mammary vein grafts in minipig. Furthermore this model should permit investigation of the pathophysiology of intimal hyperplasia and assessment of pharmacological strategies for its prevention.

## Methods

Six female Goettingen minipigs pigs (weight 53.4 ± 3 kg, age 10–12 month) underwent aortocoronary bypass grafting with mammary vein as a free transplant with cardiopulmonary bypass. Minipigs were killed after four weeks, and vein grafts were harvested. Histological and immunohistological investigation were performed with respect to morphometric analysis, endothelial damage/dysfunction, smooth muscle cells and proliferation rate. The study was approved in accordance with the ethic guidelines of the local ethics committee and federal laws. All minipigs received human care in accordance with the "Guide for the Care and Use of Laboratory Animals" as revised by the National Institutes of Health in 1985.

### Anesthesia

On the day of surgery all pigs received an intramuscular injection of 10 mg/kg azaperone (Stressnil^®^, Janssen, Neuss, Germany). Pigs were anesthetized and intubated with a combination of ketamine 10 mg/kg and xylazine 0.1 mg/kg by an intravenous injection and a prophylactic dose of antibiotic dose with 2 mg/kg cefquinome (Cobactan^®^, Intervet Deutschland GmbH, Unterschleißheim, Germany) were given.

After induction and intubation, the pigs were ventilated mechanically using a mixture of isoflurane 1.5–3 Vol%, and pure oxygen. Anaesthesia was maintained with piritramid 75 μg/kg/h (Dipidolor^®^, Janssen, Neuss, Germany) and ketamine (Ketanest^®^, Deltaselect, Dreieich, Germany) 5–8 mg/kg/h. Intravenous line was inserted in an ear vein and arterial line were inserted and in the femoral artery for continuous blood pressure monitoring.

### Operative procedure

The pigs were placed in a dorsal recumbence, and a median sternotomy from the tip of the xyphoid bone to the suprasternal notch was performed. Following placement of a sternal retractor, the left internal mammary vein was dissected free of surrounding tissue by "no touch" technique. The dissection was begun distally at the artery's bifurcation and proceeded cephaladly as far as possible into the chest cavity apex. From the distal end of the vein, a short segment was separated for further histologic and immunohistochemistry investigations.

The pericardium was opened longitudinally. Inactivation of the coagulation system necessary for the cardiopulmonary bypass was achieved by an injection of 400 IU heparin/kg (Liquemin^®^, 25000, Hoffmann-La Roche, Grenach-Wyhlen, Germany). The cardiopulmonary bypass was established via cannulation of the ascending aorta and right atrium. The body temperature was cooled to 32°C under extracorporeal circulation. Cardioplegic cardiac arrest was achieved by antegrade infusion of cold crystalloid cardiplegia (Bretschneider HTK Custodiol^®^, Köhler Chemie, Alsbad, Germany). Following cardiac arrest the left anterior descending coronary artery was prepared for. After preparing the left anterior descending coronary artery, a suited location was chosen. The internal mammary vein was connected as free graft to the artery using conventional suture technique (7/0 Prolene^®^, Ethicon, Inc., Somerville, NJ, USA). After connection of the distal anastomoses, minipigs were slowly rewarmed to 36°C. The proximal anastomoses were performed during tangential clamping of the ascending aorta while reperfusion of the beating heart on cardiopulmonary bypass. After clamp removal, the proximal left anterior descending was ligated with a suture (5/0 Prolene^®^, Ethicon, Inc., Somerville, NJ, USA) to guarantee exclusive bypass flow. Sufficient blood flow within the bypass was checked by palpation of arterial pulsation and the absence of macroscopic sudden myocardial infarction. After coming off bypass protamine was adminstrated (Protamin^®^, Hoffmann-La Roche, Grenach-Wyhlen, Germany) to neutralize the heparin dosage to 100%. Temporary substernal and intrapleural drainage tubes (28 mm, Sherwood Medical, Crawley, England) were placed and connected to continuous suction system (Redovac^®^, Sherwood Medical, Crawley, England). The chest was closed using stainless steel wires (1.75 mm, Deknatel^®^, Lübeck, Germany). With the beginning of skin suture (2/0 Prolene^®^, Ethicon, Inc., Somerville, NJ, USA) the inhalation mixture was changed to pure oxygen and the respiratory minute volume was slowly reduced until spontaneous respiration was present. General anesthesia was terminated and the minipigs were extubated upon resumption of vagal reflexes. Postoperative analgesic treatment was not performed. Before extubation the thoracic drainages were removed.

Four weeks later the minipigs were anaesthetized again in the way describe above and the chest was re-opened. After macroscopic documentation concerning the function of the mammary vein graft and examined of signs of infection, the minipigs were killed with in injection of potassium (Kaliumchlorid 7.45%, Braun, Melsungen, Germany) under anesthesia. The complete mammary vein bypass was carefully removed and perfusion-fixed 4% formaldehyde solution. The bypass was infused with fixative for 10 minutes at a pressure of 80 mmHg according to a standard protocol [10].

#### Histological techniques

##### Morphometric analysis

The diameters of the harvested grafts were measured close to the proximal anastomosis. This part of the graft was adjacent to the histological sample of the native vein.

Perfusion-fixed vein segments were embedded in paraffin, and cut transversely into 5 μm thick sections, which were stained in haematoxylin-eosin and van Giesson stain to make the elastic fibers visible. Segments of internal mammary vein grafts and ungrafted internal mammary vein wall dimensions were measured by computer-aided planimetry [Hardware: Olympus^® ^BH-2 microscope video camera, head (JVC TK-870E), computer (Victor V386A, Victor Technologies); software: SoftaOlympus DP-Soft 3.1^® ^image-analysis system (Olympus Europe Holding GmbH, Hamburg, Germany)]. Wall thickness of intima was measured from the subendothelial basal lamina to the internal elastic lamina, from where media extends to the adventitial layer in four regions of interests with respect of the cardinal points of a compass (*North*, *East*, *South*, *West*). Luminal areas were also determined from these histological slices.

##### Immunohistochemistry and cell proliferation analysis

Proliferation of the intimal cells was assessed using immunhistochemical reaction with monoclonal antigen [11,12] (Ki-67, Dako GmbH, Hamburg, Germany). Smooth muscle cells proliferation throughout the new intima were identified by using monoclonal antibodies directed against α-smooth-muscle-actin (clone 1A4, Dako GmbH, Hamburg, Germany). Vein sections were also stained with rabbit anti-human von Willebrand human factor (vWF, Dako GmbH, Hamburg, Germany) to identify dysfunction of endothelial cells.

### Statistical analysis

All date were tested for normal distribution with the Kolmogorov-Smirnov-test and then analyzed further with parametric methods as indicated using commercial statistic software (Statistica 5.1.^®^, StatSoft Inc., Tulsa, OK, USA). Comparison between ungrafted vein and graft-vein was carried out with a Mann-Whitney U-Test. Results were considered significant if *p *was less than 0.05.

## Results

Eight animals entered into the protocol, six were used in the final analysis. The initial two animals developed intractable ventricular fibrillation during coronary occlusion stage or immediately afterwards and it was impossible to defibrillate the heart using electric shock. There were killed, humanely. In the final analysis no animal died prematurely. None developed incision infection.

The native veins were investigated after harvesting immediately. Grafts of the mammaria vein were investigated four weeks after coronary artery bypass grafting. None of the studied minipigs showed cardiac events (i.e. infarction, arrhythmic, sudden death). Luminal area differed not between native vein (1.50–12.07(4.25) mm^2^) and graft (0.03–26.08(0.96) mm^2^).

**Figure 1:**
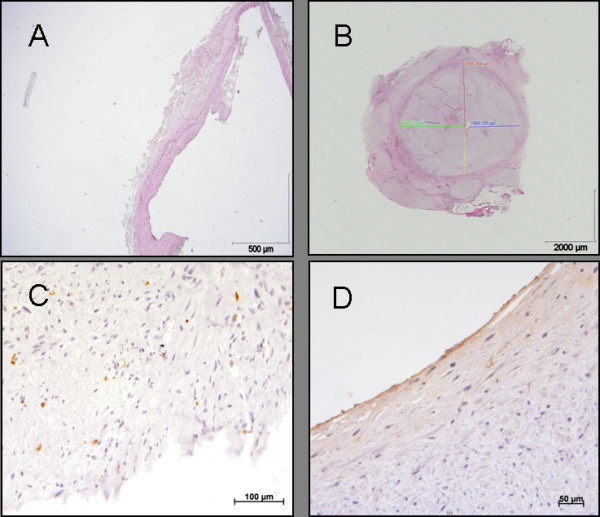
Histological appearance of an ungrafted internal mammaria vein (A), a representative grafted mammaria vein after four weeks, with measurements of intimal hyperplasia in respect of the cardinal points of a compass (B), proliferating intimal cells in brown (proliferationsmarker KI 67) (C), dysfunction of endothelial cells (vWF) (D).

Histological section demonstrated hyperplasia of new intima. Immunohistochemistry showed a proliferation of the intimal cells. This was confirmed by massive accumulation of smooth muscle cells with a high proliferation rate and endothelial perturbation and an increase of von Willebrand factor as an indictor of endothelial dysfunction (Figure 1). Significant intimal hyperplasia of the grafted vein compared to the native vein was found by an increase of wall thickness in the four regions of interests (Table 1).

**Table 1:** Dimensional analysis. Luminal area and thickness of the native mammaria vein and grafted mammaria vein. Thickness was measured in four regions of interest with respect to the cardinal of the compass and as mean of the four regions of interest.

**Mammaria vein**	**Native**	**Graft**	***p *value**
Luminal area (mm^2^)	1.50–12.07(4.25)	0.03–26.08(0.96)	*n.s*.
Intima thickness			
*North *(μm)	84–221(120)	424–2469(1882)	*0.001*
*East *(μm)	74–232(146)	839–2377(1946)	*0.001*
*South *(μm)	74–253(130)	707–2372(1632)	*0.001*
*West *(μm)	61–246(142)	654–2021(1375)	*0.001*
Mean (μm)	61–253(134)	424–2469(1733)	*0.001*

## Discussion

The intent of this study was to describe a new animal model with respect to accelerated intimal hyperplasia in aortocoronary vein grafts. Intimal hyperplasia is one of the dominant mechanisms underlying the occlusion of veins used in coronary artery bypass surgery. It is driven by cell proliferation and migration, and elaboration of extracellular matrix, predisposes to atheromatous thickening of the venous graft, ulceration of the endothelium and thrombosis [2]. These mechanisms contribute to the high incidence of occlusion of vein grafts used in coronary artery bypass surgery.

In our study we chose internal mammary vein grafted as a free transplant to ascending aorta to left anterior descending coronary artery. This operative procedure corresponds with routine aortocoronary bypass surgery with veins in humans. As the diameter of the used internal mammary vein is more likely comparable to the diameter of left anterior descending artery, it simplifies aortocoronary bypass surgery. We found an accelerated intimal hyperplasia in vein grafts after four weeks indicated by morphometric analysis, endothelial damage/dysfunction markers, and proliferation of smooth muscle cells. Smooth muscle cell proliferation is a key event in the development of atherosclerosis, restenosis and intimal hyperplasia [13,14].

This is in accord with theoretical predictions and the experimental work of others who investigated intima hyperplasia in animal models. Numerous studies have documented intimal hyperplasia in vein grafts in small and large animals [4-8,15].

In mice vena cava were inserted into carotid artery [15,16]. In rats the ileolumbar vein and inferior vena cava were placed into abdominal aorta and superficial epigastric vein into femoral artery [17,18]. The jugular vein was inserted into carotid artery in rabbits [19,20]. In canines jugular vein were inserted into carotid artery, saphenous vein were grafted into coronary artery, femoral vein and jugular vein were placed in femoral artery [8,21-23]. Numerous pig models are described for saphenous vein or jugular vein interposition into carotid artery [4-7].

However all of these models do not investigate intimal hyperplasia during aortocoronary bypass surgery. The present model complements other *in vivo *models of venous intimal hyperplasia.

The advantage of our model is the more human like wall thickness of the vein graft compared with thin wall of small animal veins. The anastomosis of venous segments directly into the arterial circulation in small animals represents a technically demanding model, requiring microsurgical expertise [24,25].

We chose a minipig model to show a model for cardiovascular research in respect of intimal hyperplasia in mammaria vein grafts. Pigs are one of the readily available species in which cardiovascular anatomy and physiology resembles humans. There are many similarities between the species. In humans and pigs, size and distribution of coronary arteries are similar [26] with blood flow through the right and left anterior descending coronary arteries supplying nearly 80% of the myocardium in pigs. Standard operative cardiac techniques can be applied in the minipig using the same equipment used for human procedures. Size of the vessels and heart of minipig make them ideal for developing and testing intravascular or extravascular device prototypes similar in size and configuration to the model that may be used in humans. Therefore, pigs are widely used as a model of intimal hyperplasia, and they have become the model of choice for studies in cardiac interventional procedures.

No pig model have already shown vein graft disease of internal mammary vein which were grafted in a reversed manner from the ascending aorta to the left anterior descending coronary artery followed from ligating the proximal left anterior descending coronary. We note that our model focuses primarily on the venous intimal hyperplasia. Furthermore our model may be useful for studying the pathology of vein graft disease and test therapeutic approaches. Thrombosis, inflammatory cell mirgration and neointimal hyperplasia in arterialized veins can be studied in this presented model.

A limitation to this study is the small number of experimental animals. Further studies are therefore necessary. Furthermore, this model exacts substantial financial cost and other requirements involved in large animal research. Small animal models are cheaper, and proper animal care is easier available compared with large animals.

## Conclusion

In conclusion, this new model is suitable for investigations in accelerated intimal hyperplasia in aortocoronary internal mammary vein grafts at minipigs. It may support the investigation of pathophysiology of aortocoronary vein graft intimal hyperplasia as well as therapeutic approaches.

## Competing interests

The authors declare that they have no competing interests.

## Authors' contributions

AP had helped with surgical techniques, performed data, analysis, statistics, graphics, and wrorte the paper. JH helped with data interpretation and helped to draft the manuskript. HD, JS and TS and did the surgery on the animals. VD performed the histological investigations. FS co-wrote the manuscript and added important comments to the paper. All authors read and approved the final manucript.
